# Disease Related Knowledge Summarization Based on Deep Graph Search

**DOI:** 10.1155/2015/428195

**Published:** 2015-08-25

**Authors:** Xiaofang Wu, Zhihao Yang, ZhiHeng Li, Hongfei Lin, Jian Wang

**Affiliations:** College of Computer Science and Technology, Dalian University of Technology, Dalian 116024, China

## Abstract

The volume of published biomedical literature on disease related knowledge is expanding rapidly. Traditional information retrieval (IR) techniques, when applied to large databases such as PubMed, often return large, unmanageable lists of citations that do not fulfill the searcher's information needs. In this paper, we present an approach to automatically construct disease related knowledge summarization from biomedical literature. In this approach, firstly Kullback-Leibler Divergence combined with mutual information metric is used to extract disease salient information. Then deep search based on depth first search (DFS) is applied to find hidden (indirect) relations between biomedical entities. Finally random walk algorithm is exploited to filter out the weak relations. The experimental results show that our approach achieves a precision of 60% and a recall of 61% on salient information extraction for *Carcinoma of bladder* and outperforms the method of Combo.

## 1. Introduction

Biomedical literature is growing rapidly in recent decades. Up till now, the number of papers indexed in PubMed is over 23 million. The continued growth of biomedical literature brings about great challenges to traditional information retrieval (IR) techniques. Standard search techniques, when applied to large databases such as PubMed, often return large, unmanageable lists of citations, which makes it difficult for clinical experts to find the salient information they need. Therefore, effective biomedical literature retrieval, especially finding salient information related to disease from the biomedical literature, is greatly helpful in terms of clinical trials and patient treatment.

Abstraction summarization offers an attractive alternative for managing citations resulting from MEDLINE searches. Many biomedical text summarization methods using information retrieval (IR) techniques together with domain-specific resources have been proposed to extract relevant sentences from documents [1, 2]. Luhn developed a text summarization system which selects relevant sentences and generates text abstracts from biomedical literature based on term frequencies [1]. Reeve et al. used the frequency of domain concepts to identify important parts of a paper and then use the resulting concept chains to extract candidate sentences [2]. However, these methods for similarity calculation are only at a word or concept level rather than a semantic level, since they measure the similarity merely based on the common words or concepts in the query and sentence, which is the major difficulty that limits the performance improvement for text summarization system.

Recently, more IE-based methods based on the Semantic MEDLINE have been proposed [3–10]. Compared with the classical IR-based methods, these methods can extract semantic knowledge from biomedical texts in a higher level. Fiszman et al. applied the technique of information extraction (IE) to extract the entities and relations that are most relevant to a given biological concept from MEDLINE records and generate a “semantic level” summary for each concept [3]. More biomedical researches of IE became popular based on the Semantic MEDLINE. Compared to the classical IR-based methods, these methods can extract semantic knowledge from biomedical texts in a higher level. Shang et al. presented a method for generating text summary for a given biomedical concept from multiple documents based on semantic relation extraction using IR and IE [4]. Kilicoglu et al. developed a web application, called Semantic MEDLINE, which integrates PubMed with natural language processing, automatic summarization, visualization, and interconnections among multiple sources of relevant biomedical information [5]. Several years later, they presented SemMedDB, a repository of semantic predications (subject-predicate-object triples) extracted from the entire set of PubMed citations [6]. Lately, many researches have been made on this biomedical semantic database. Workman and Hurdle presented the Combo algorithm to extract the genetic predicates for a particular disease, which outperformed a conventional summarization schema based on Semantic MEDLINE summarization in a genetic database curation [7]. Later, they proposed a novel dynamic summarization method in identifying decision support data [8]. Hristovski et al. proposed an innovative methodology for biomedical QA, implemented as a search in the semantic database [9].

However, these methods can only extract the direct relation between biomedical entities and, therefore, cannot obtain deep comprehensive information related to the seed topic. To solve the problem, we present a depth first search (DFS) based knowledge summarization approach, which can find not only direct relations between biomedical entities but also their hidden (indirect) relations. In this approach, a novel algorithm of salient information summarization, KM, is used to obtain the direct relations between disease and genes. Then DFS is applied to extract indirect relations between disease and other related entities. At last, the weak relations are filtered out with the random walk algorithm. The approach is applied to automatically construct the knowledge summarization of the disease* Carcinoma of bladder* from biomedical literature and the experimental results verify its effectiveness.

## 2. Method

**Figure 1:**
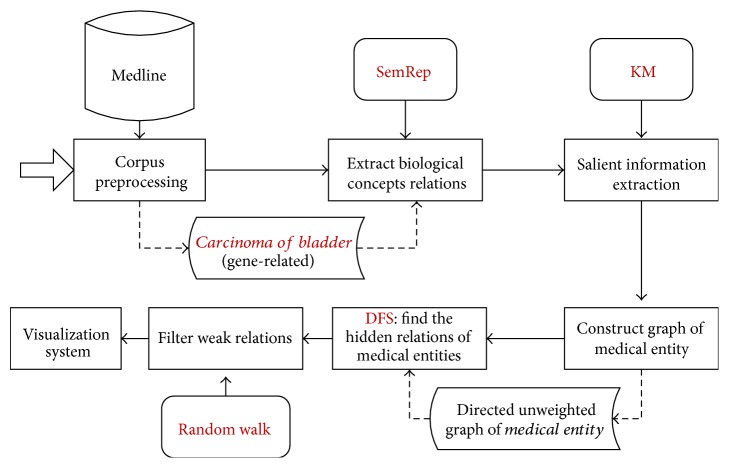
The framework of our method.

Our method consists of four major steps as shown in Figure 1. (1) Semantic relations are extracted from the sentence by semantic knowledge representation tool SemRep [11, 12]. (2) The relations most relevant to the seed topic are selected with the summarization algorithm based on Kullback-Leibler Divergence (KLD) [13] and Mutual Information [14, 15] (KM). (3) The hidden relations are extracted using deep search based on DFS from the directed unweighted graph of biomedical entities. (4) The weak relations are filtered out with the random walk algorithm and the final results are visualized.

### 2.1. Dataset

In our experiments, the disease name* Carcinoma of bladder* was chosen as the seed topic to present our work in salient information extraction. The dataset of the seed topic was downloaded from PubMed by the following query:

 (“2003/01/01” [Publication Date]: “2013/07/31” [Publication Date]) AND (Urinary Bladder Neoplasms/genetics [majr] AND Urinary Bladder Neoplasms/etiology [majr]) AND English [la] AND humans [mh].

The search query focuses on the genetic etiology (the point-of-view) of human bladder cancer (the seed topic) with a more than ten years span.

### 2.2. Corpus Preprocessing

The downloaded corpus is processed to the semantic predications provided by SemRep, a natural language processing tool based on the rule to identify relationship in the MEDLINE documents. SemRep integrates MetaMap standardized conceptual entity and connects the concept of different entities through predicate relations. In addition, semantic type of SemRep term is defined for each entity, making it convenient for feature selection. The output of SemRep extraction is based on UMLS rules. The original output contains many terms, but we mainly use the name of the entity, semantic type, and predicate relations.

For sentence “miRNA expression arrays and individual qPCR were used to identify and confirm miRNAs that were downregulated in malignant urothelial cells (RT4, 5637 and J82) when compared to primary, non-malignant urothelial cells (HUEPC)”, the relation extracted by SemRep is as follows:

 SE|23867826||ab|4|relation|3|1|C1101610|MicroRNAs|bacs,nnon|bacs|||miRNAs||||1000|972|978|VERB|DISRUPTS||995|1008|5|1|C0227599|Transitional epithelial cell of urinary bladder|cell|cell|||urothelial cells||||890|1022|1038.

Our study focuses on the triple terms as follows:

 MicroRNAs|bacs|DISRUPTS|Transitional epithelial cell of urinary bladder|cell.

The relation structure is (concept 1 | semantic type, predicate, concept 2 | semantic type). Concept 1 and concept 2 are the two biological entities that can be found in UMLS (Unified Medical Language System) Metathesaurus. Each concept consists of the standardization of the concept representation, concept unique identifier, and semantic type. Predicate is an indicator of the relation type in UMLS Semantic Network and 54 predicates are defined in the semantic network of UMLS (e.g., DISRUPTS is one of them).

### 2.3. Salient Information Summarization

In this task, a semantic predication such as “TP53 gene | ASSOCIATED_WITH | Carcinoma of bladder” is desirable, because it conveys the salient information to the work of annotating gene and disease process information in a database like OMIM or GHR. The semantic predicate “DELETION | COEXISTS_WITH | Carcinoma of bladder” is not desirable, because it offers no information addressing pathologic changes in disease development for biomedical researchers [7].

In this study, we present a new summarization algorithm that identifies the salient SemRep output. The algorithm is evaluated by comparing its performance with that of Combo [7], which is used in a genetic database curation task. Combo uses three metrics, namely, KLD [13], *R*log⁡*F* [16], and PredScal [7]. KLD scores express a proportional relationship among predicates across the entire dataset, while *R*log⁡*F* values express a binding between a single predicate and its associated semantic types. Then a scaling function PredScal is used to scale *R*log⁡*F* values according to the spatial proportions of predicates in a given dataset. In our method, two metrics, KLD and MI, are combined to extract information. KLD calculates the significance only for one entity in two different datasets, and MI measures the reciprocity for a pair of entities. Our method highlights the importance of the predication by combining KLD and MI. Since these two metrics are both entropy related, the combination of them can hopefully improve the performance of the salient SemRep extraction, which is also verified by our experimental results.

#### 2.3.1. Kullback-Leibler Divergence

Kullback-Leibler Divergence (KLD) is known as the relative entropy in information theory to measure the relative distance between two probability distributions. It is typically used in measuring the similarity of the two models. If two models are completely the same, the KLD score is 0. In the field of IR, a special feature distributing in the two data sets can be seen as two different distribution models. We select part of data set *P* from a large data set *Q*. In the two datasets, *P* and *Q*, the importance of a feature in the small data set can be measured by the value of KLD. A large value of KLD indicates that there is a large difference in the characteristics of small data set and the distribution of large data sets. Therefore, we can conclude that this feature is a salient role to the data set *P*. Consider

(1) $$\begin{array}{c} D\left(\;P||Q\;\right)=\sum P\left(\;x\;\right)\log_{2}\left(\;\frac{P\left(x\right)}{Q\left(x\right)}\;\right), \end{array}$$

where *x* represents the relative frequency of each unique predicate in each distribution. In our study, we choose the distribution of SemRep predicates resulting from a PubMed query that expresses the seed topic, as distribution *P*, with a large dataset of predicates of all SemRep predicates between January 1, 1999, and July 31, 2013, as distribution *Q*. The individual KLD calculation (before summing) indicates the prevalence of each predicate in distribution *P*. Semantic predications in both distributions are limited to those containing a certain UMLS Metathesaurus seed topic.

#### 2.3.2. Mutual Information

Mutual information (MI) is a common analysis method of calculating linguistic model, measuring reciprocity between the two objects [16]. In processing discrimination issues, MI is used to measure the object characteristics for one specific subject. Since MI does not need to make any assumptions on the feature of the words and the relationship between words categories, it is very suitable for text classification with prospective job of characteristics and categories.

In the dataset, certain semantic types and relation predicates are associated with each other in the statistic characteristic. In a particular search process for a seed topic entity, we can obtain many relation predicates related to the seed topic and semantic types related to a relation predicate. For example, since our study aims to obtain the genes related to the selected disease topic,* gngm* is the most related semantic type. If an algorithm assigns a high score to semantic type* gngm*, it is deemed to be effective in this step of the information extraction. Since MI can measure the reciprocity between semantic types and relation predicates, it is applied in our study to filter out the semantic types and relation predicates. MI is defined as follows:

(2) $$\begin{array}{c} I\left(\;x;y\;\right)=\underset{x,y}{\sum }p\left(\;x,y\;\right)\log\frac{p\left(x,y\right)}{p\left(x\right)p\left(y\right)}, \end{array}$$

where *x* represents the semantic type of the entity and *y* represents the relation predicate. For every *x* and *y*, we use MI to calculate potential relationships between them. We figure out the semantic type probability *p*(*x*) and the relation predicate probability *p*(*y*) from the data set, using them in the mutual information process.

#### 2.3.3. Semantic Association Generation

KLD is effective in the relation predicate extraction, and MI can extract the reciprocity between semantic type and predicate. Therefore, a novel algorithm combining KLD and MI, called KM, is brought up to extract salient relations among kinds of entities:

(3) $$\begin{array}{c} KM\left(\;e_{1},e_{2}\;\right)=KLD*MI, \end{array}$$

where *e*_1_ represents the seed disease topic and *e*_2_ represents the entity related to *e*_1_. KLD represents the score of relation predicate between *e*_1_ and *e*_2_. MI represents the score of relation predicate and the semantic type of *e*_2_.

### 2.4. Hidden Relation Extraction

#### 2.4.1. Deep Graph Search

As discussed in previous section, we use the KM algorithm to rank the pairs of semantic types and relation predicates and retain the most important ordered pairs for the seed topic. Then the direct relations between diseases and genes can be obtained from the selected relation predicates and semantic types. However, there could be many hidden relations among the biomedical entities (such as drugs, proteins, genes, etc.) in the biomedical texts. These hidden relations are constructed by searching the direct relation between medical entities extracted by the KM method. The extraction of these hidden relations can significantly promote the development of new drugs and provide new ideas in medical diagnosis.

Then how can one discover the hidden relations between two entities? Wilkowski et al. presented a methodology to decompose ABC model to AnC model using semantic representations and graph algorithms [17]. Inspired by their AnC model, we split AnC to A*r*_1_B*r*_2_C⋯AnC with deep graph search accomplished by the DFS algorithm of graph theory [18], where *r* is relation predicate. The details are introduced as follows.

Firstly, a direct unweighted graph consisting of nodes and arcs is constructed. A node in the graph represents a biomedical entity and an arc is a directed edge from one entity to another. Since many kinds of relation predicates (such as ASSOCIATED_WITH, PART_OF, and ISA) exist between the entities, they are also regarded as nodes. As a consequence, there would be many paths between two biomedical entities. However, some high frequency general entities such as “GENE” and “PATIENTS” with large degree usually have high transition probability and similarity with other nodes and appear among the top *N* ranking nodes. Therefore, we introduce the inverse document frequency, IDF_*i*_, to filter out these general entities in our original corpus before DFS is executed [19]. Consider

(4) $$\begin{array}{c} IDF_{i}=\log\left(\;\frac{D}{D_{i}}\;\right), \end{array}$$

where *D* is the number of different documents in the corpus. *D*_*i*_ is the document frequency of entity *i*. The nodes are ranked by the weight of inverse document frequency.

Secondly, all the paths between any ordered pairs are found with the method of DFS. However, since there are nearly 3,000 nodes and 26,000 arcs in the graph, the time complexity for deep search is too high to afford. Therefore, we restrict the path lengths to depth 4 and depth 6 since the relation predicates are also nodes; the lengths of path from one entity to another can only be 2, 4, 6, 8,…. That is to say, the pair of predicate and entity is two different nodes in the graph, and the graph depth increases by 2 for each step forward. For example, in the predicate* Carcinoma of bladder*→*AFFECTS*→*Smoker*→*PREDISPOSES*→*Chromosomal Instability*, there are four nodes followed by the starting node* Carcinoma of bladder* for depth 4.

#### 2.4.2. Weak Relation Filtering

Numerous paths from one entity to others are extracted with the DFS method. However, not all the paths are salient for each entity. Therefore, we need to filter out the entities which are weakly related to the current entity. Bordino et al. performed a lazy random walk with restart to retrieve entity recommendations from the networks [20]. Compared to standard web search engines, their method provides novel results from the datasets. Inspired by their work, we apply the random walk algorithm [21, 22] to select the salient relations among all kinds of entities.

In our method, the random walk algorithm shown in Algorithm 1 is used to rank the relations according to the random access probability. This process retains the paths whose destination node is one of the top *N* entities which have higher transition probability from the current entity.

**Algorithm 1 (random walk algorithm).**

  

*Input*

 Directed weighted graph *G* = (*V*, *E*);
 Starting node *n*;
 Restart probability *α*.

*Output*

 Stationary vector for a Random Walk starting at *n*.

*Procedures*

(1) Let *s* be the restart vector with all its entries initialized to 0 except a 1 for the entry denoted by *n*;
(2) Let *M* be the row normalized adjacency (transition) matrix defined by *G*;
(3) Initialize *x*≔*s*;
(4) While (*x* has not converged)
  (5) $$\begin{array}{c} x≔\alpha s+\left(\;1-\alpha \;\right)M^{T}x; \end{array}$$
(5) Output *x*.

First of all, we define the weights of arcs as the frequency of directed cooccurrence of two entities. For two entities, *j* and *s*, *C*_*js*_ denotes the directed cooccurrence frequency of *j* to *s* in the corpus of all directed relations. Considering that the random walk is an iterative algorithm which has many steps before convergence (from step 0 to step *t*), the weight of arc between *j* and *s* from step *t* to step *t* + 1 is defined as *P*_*t*+1∣*t*_(*s*∣*j*). Consider

(6) $$\begin{array}{c} P_{t+1\;|\;t}\left(\;s\;|\;j\;\right)=\left\{\begin{array}{cc} \left(1-\alpha \right)\frac{\lambda_{js}C_{js}}{\sum_{i}\lambda_{ji}C_{ji}} & \forall s\neq j\\ \alpha & s=j, \end{array}\right. \end{array}$$

where *C*_*ji*_ is the cooccurrence degree from node *j* to *i*. Let the self-transition probability be *α* (also called the restart probability). It allows random walk to stay in place and reinforces the importance of the starting point by slowing diffusion to other nodes. *λ*_*ji*_ is the KM coefficient of the relation category corresponding to the arc from *j* to *i*.

Then, we calculate the one-step transition probabilities as a matrix *M* whose *j*th row and *s*th column element is *P*_*t*+1∣*t*_(*s*∣*j*). The random walk algorithm could be described as a process like this: we compute the transition probability from node *j* to *s* in *t* steps denoted by *P*_*t*∣0_(*s*∣*j*) and equal to [*M* ^*T*^]_*js*_. Then the probabilities of all paths between the two nodes should be accumulated. So, if there are many paths between two nodes, the transition probability will be high. The parameter *α* in our experiment is set to 0.7, which is an empirical value [22]. As convergence criterion, we check whether the weight difference between two iterative steps is less than the threshold (10^−6^), or we can just stop the algorithm after a specific number of iterations (e.g., 50). The optimal parameters are determined through experiments.

Finally, we obtain two matrixes: the transition matrix *R*_*n*×*n*_ which records the transition probability between each node pair and the similarity matrix *S*_*n*×*n*_ which records the similarity between each node pair.

## 3. Experimental Results and Discussion

### 3.1. KM Performance Analysis

#### 3.1.1. Entity Predication Extraction

In our experiments, 1,293 Medline citations focused on genetic etiology of* bladder cancer* are used. SemRep processes these citations and returns 8,241 semantic predications. Then KM algorithm is applied in disease-gene predication. More relations (among genes, proteins, diseases, symptoms, etc.) are extracted by deep graph search.

Similar to the Combo method, our salient summarization method is applied in the disease-gene predication. With* Carcinoma of bladder* as the seed topic, KLD method extracts the relation predicates in the data set. Relation predicates with KLD scores larger than 0.01 (which is determined through experiments) are displayed in Table 1. It can be seen that* ASSOCIATED_WITH* is the most salient relation about* Carcinoma of bladder*. MI here is used as a measure of the cooccurrence information between the relation predicates and the entity semantic types. MI scores semantic types related to the relation predicates. The entity semantic types and the relation predicate salient in the data set are chosen from the MI results.

All novel semantic predications including KLD top relations and the MI ranking results are extracted as salient output. We identify the salient output using the most prominent predicates and semantic types in KLD and MI computation and then extract all genetic entities serving as subject or object arguments in the salient output.

The results of KM are shown in Table 3. The highest-ranking semantic type* gngm* (the abbreviation of* Gene or Genome*; the full names for the semantic types are shown in Table 2) is the semantic type for gene which we need. That is to say,* ASSOCIATED_WITH* is the most relevant relation predicate between the diseases and genes. To compare the performance of KM algorithm with that of Combo, we reproduce the Combo algorithm as the control test in our ten years' corpus. The result of Combo is shown in Table 4. The ranking of* gngm* (first) in the KM results is superior to that (second) in Combo results. In addition, though the scores of the two algorithms are with different standards, it can be seen that* gngm ASSOCIATED_WITH* ranks first and has a KM score of 0.036137 which is much higher than the others. While in Table 4,* gngm ASSOCIATED_WITH* ranks the second and its score is almost the same with the third ranking* celc PART_OF*. That shows KM has a better performance in extracting the entity semantic types and relation predicates than Combo.

#### 3.1.2. Performance Evaluation

To evaluate the performance of KM method, the performance of the topic-related genetic entity extraction is explored. Similar to the method used in [7], the extracted genetic entity names are normalized to the associated gene names in Entrez Gene and compared to a reference standard of genes implicated in* bladder cancer* development in the OMIM and GHR records.

**Figure 2:**
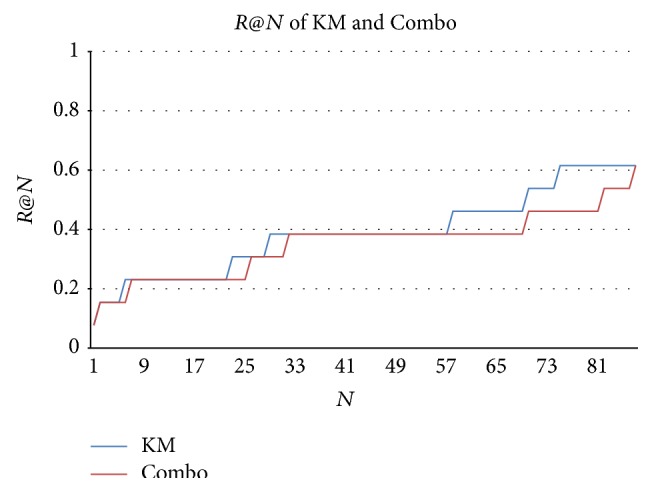
*R*@*N* of KM and Combo. *R*@*N* is the recall of top *N* samples in the ranking; *N* is the number of samples.

To compare with Combo, the same metrics, namely, recall, precision, and *F*-score (defined as *F* = (2*PR*)/(*P* + *R*) where *P* denotes precision and *R* recall) used in [7], are employed to evaluate KM's performance. Recalls are calculated by comparing outputs to the reference standard of genes noted in relevant GHR and OMIM records. The reference standard provides a list of genes whose values have already been confirmed within the task of secondary genetic database curation, because GHR and OMIM curators have annotated their potential roles in bladder cancer development. In addition, *R*@*N*, the recall in the top *N* results, is used to evaluate the recall performance in only the topmost results returned by different methods. The results of the reference standard analysis are listed in Table 5. KM and Combo methods achieve the same recall (61%) since they are both based on semantic predications generated by the SemRep, while these predications only include the eight genes of the total thirteen genes (61%). However, the genes summarized by KM have better rankings than those of Combo as shown in Figure 2. The mean average precision (MAP) of KM (39.46%) is higher than that of Combo (37.52%).

The metric precision indicates the ratio of the truly relevant genes about* Carcinoma of bladder* which are found by the method. To assess validity (true positive or false positive status) for the additional genes, Genes into Reference (GeneRIF) notations in relevant NCBI Entrez Gene records are reviewed for disease process implication [7]. If the relevant Entrez Gene record does not contain applicable GeneRIFs but otherwise notes* bladder cancer* association in NCBI PubMed, the gene is assigned true positive status. Precision is evaluated by taking the previously established true positive findings into account with the additional genes. Similar to *R*@*N*, the precision in the top *N* results, *P*@*N* is used to evaluate the precision performance in only the topmost results returned by different methods.

**Figure 3:**
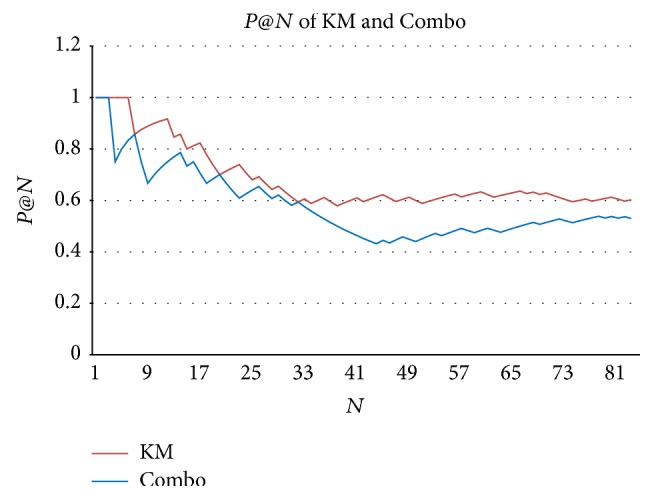
*P*@*N* of KM and Combo. *P*@*N* is the precision of top *N* samples in the ranking. *N* is the number of samples.

KM returned 84 results, among which 50 genes are related to* Carcinoma of bladder* in PubMed and regarded as true positive (TP). The precision of KM is 60%. In 95 results returned by Combo, 49 genes are true positive (TP), and the precision of Combo is 53%. The KM method achieves a better precision than the Combo method. In addition, as shown in Figure 3, the *P*@*N* scores of KM are much higher than those of Combo. All these show that the precision performance of KM is better than that of Combo.

In addition, we calculate the *F*-scores, the harmonic mean of precision and recall, to assess the overall performance. The KM method achieves an *F*-score of 61%, which is better than that of the Combo method (57%).

To further assess the performance of the KM algorithm, we applied KM and Combo in summarizing* Parkinson Disease* SemRep data. The PubMed query is as follows:

 (“2003/01/01” [Publication Date]: “2013/07/31” [Publication Date]) AND (Parkinson Disease/genetics [majr] AND Parkinson Disease/etiology [majr]) AND English [la] AND humans [mh].

The query returned 2,159 abstracts focusing on genetic etiology of Parkinson Disease. Then SemRep processed these abstracts, resulting in 9,171 semantic predications. Recalls are calculated by reference to the standard of genes noted in relevant GHR and OMIM records. The results of the reference standard analysis are listed in Table 6. As with the results of* bladder cancer*, although the recalls of KM and Combo are both 27%, the genes summarized by KM have better rankings than those of Combo. In addition, the MAP of KM (62.66%) is also higher than that of Combo (27.86%).

The precision is the metric of the truly relevant genes about* Parkinson Disease*. To assess validity for the additional genes, we marked the top 100 results returned by two methods referring to PubMed. There are 80 genes related to* Parkinson Disease* returned by KM, while only 53 genes are returned by Combo. That is to say, KM achieves a higher precision (80%) than Combo does (53%) in the top 100* Parkinson Disease* results.

We also calculate the *F*-scores to assess the overall performance on* Parkinson Disease*. The *F*-score of KM (40.37%) is better than that of the Combo method (35.77%).

### 3.2. Hidden Relation Extraction

#### 3.2.1. Deep Graph Search Analysis

The KM method completes the extraction of entity semantic types and relation predicates. Then a deep search method is applied to automatically decompose the KM results using semantic representations and graph algorithms. Deep extraction helps to understand interactions between entities. Our method executes deep search for the new discovery based on entity relationship diagram.

In our experiments, depth 4 and 6 are selected as the stopping conditions for the DFS algorithm, respectively. At depth 4, 1,896 semantic predications are retained after being filtered by IDF scores, and it costs a maximum running time of three minutes to generate the reachable relation. At depth 6, 92,849 semantic predications are retained, while the maximum running time increases to 113 minutes. Empirically, our experiments suggest that many associations are returned by the algorithm, while few associations will be returned beyond the maximum depth of 6. Hence, we also report here on associations generated to the depth 6.

#### 3.2.2. Weak Relation Filtering

In our study, the random walk algorithm is used to calculate the similarity of biomedical entities in the direct unweighted graph. Then the weak relations are filtered out by the similarity threshold of random walk, which ensures that the hidden relations or paths are more available. Transfer probability (set to 0.7 as discussed in Section 2.4) is a restart probability in the random walk process. According to the transfer probability scores, the important relations are selected by the ranking. We analyze the results and obtain a lot of important entities that did not appear in the KM extraction results, including genes, drugs, proteins, chemical elements, and symptoms.

Our scoring method ranks the entities based on the stationary distribution of the random walk described above. We obtain 240 semantic predications for depth 4 and 4,679 semantic predications for depth 6 by the random walk filtering. Top 10 predications of depth 4 are shown in Box 1. For example, we analyze the returned relations* Carcinoma of bladder*→*AFFECTS*→*Smoker*→*PREDISPOSES*→*Chromosomal Instability *and* Carcinoma of bladder*→*AFFECTS*→*Dysplasia*→*COEXISTS_WITH*→*HRAS gene* in Box 1 using PubMed as the reference. There are 29 records returned about* Carcinoma of bladder* and* Smoker* and 415 records returned for* Dysplasia* and* Carcinoma of bladder*.

**Figure 4:**
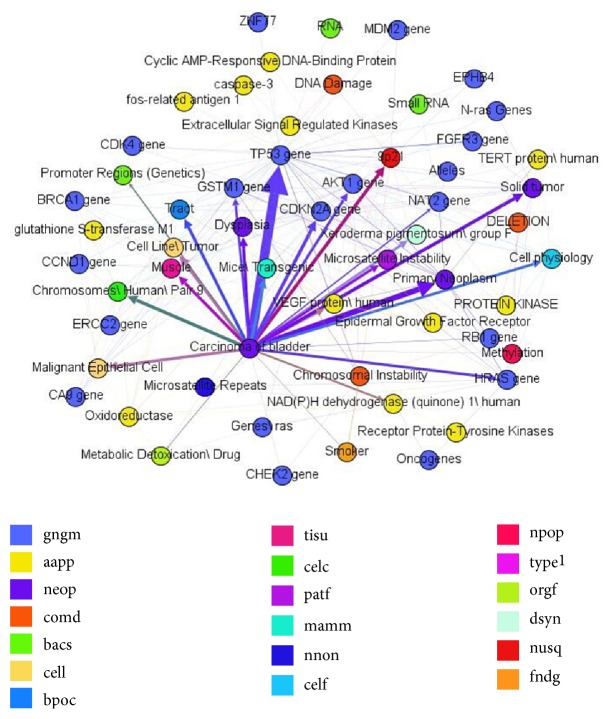
Disease information extracted by random walk with depth 4.

Box 1 is a part of the relations returned by random walk with depth 4. The whole relations network is shown in Figure 4, which illustrates the information of the seed topic* Carcinoma of bladder*, which displays genes, drugs, proteins, chemical elements, or symptoms in different colors. The degree of the relation strength is shown by thick or thin edges.

**Figure 5:**
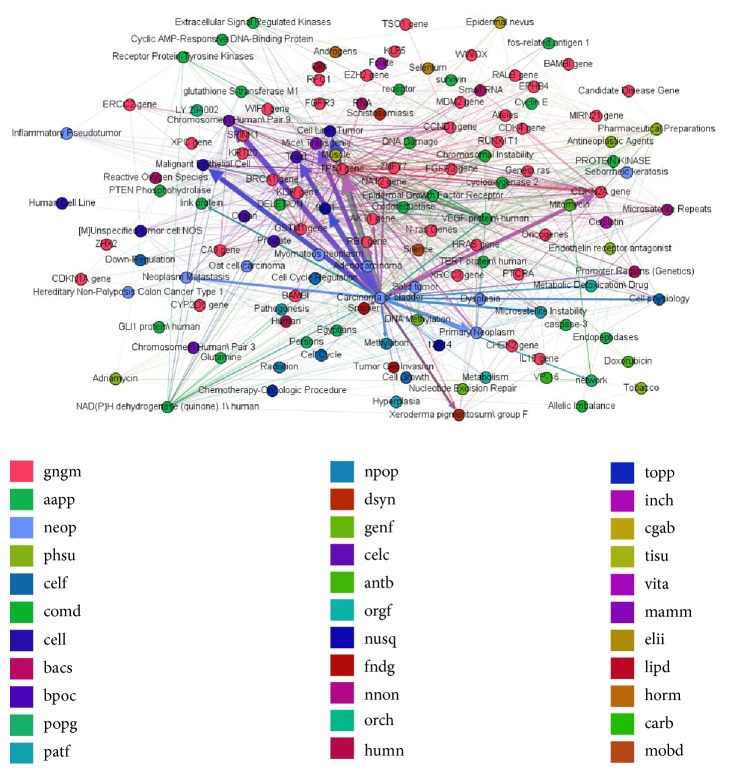
Disease information extracted by random walk with depth 6.

Similarly, top 10 predications of depth 6 are listed in Box 2. Among others, the returned relation* Carcinoma of bladder*→*COEXISTS_WITH*→*CDKN2A gene*→*ISA*→*Retinoids*→*STIMULATES*→*TP53 gene* shows that* retinoids* are related to* Carcinoma of bladder* by stimulating* TP53 gene*. The relation can also be verified with reference to PubMed. The whole relations returned by random walk with depth 6 are shown in Figure 5. It illustrates more sufficient disease information among all kinds of entities.

To highlight the performance of the AnC model based on KM, the DFS process generated by the cooccurrence pairs from SemRep was carried out. Without the KM summarization, we take* Parkinson Disease* as the start node and the entities most related to* Parkinson Disease* by the cooccurrence as the end nodes of the DFS. The DFS outputs are scored by random walk. The top 10 results of depth 4 and 6 are shown in Boxes 3 and 4, respectively. We can see that some general terms, for example,* Patients*,* Neoplasms*,* Malignant Neoplasms*,* Carcinoma,* and* Transitional Cell*, appear frequently in the results because these words have high frequency in the cooccurrence with* Parkinson Disease*; but these relations cannot provide useful information in disease analysis and, therefore, are of little value. To sum up, comparing the results of KM based and cooccurrence based AnC models, the performance of former is much better than that of the latter.

## 4. Conclusions and Future Work

In this paper, we present an approach to automatically construct disease related knowledge summarization from biomedical literature, which can find not only direct relations between biomedical entities but also their hidden relations. Firstly, the approach applies the KM method to extract salient genetics information from disease-gene predications. The method is evaluated with a previously established reference standard and achieves an *F*-score of 61%, which is better than that of the Combo method (57%).

Secondly, in our approach, a graph-based methodology based Semantic MEDLINE is presented to mine hidden relations between biomedical entities. Then excessive outputs of deep graph search are filtered out by the random walk algorithm, which uses the transfer probability as a criterion to select relations between entities. Our experimental results show that the method of deep graph search can obtain hidden salient relations.

In the future, we will further improve the performance of our method and construct a comprehensive biomedical information summarization system which can effectively extract hidden relations among all kinds of entities from biomedical literature.

## Figures and Tables

**Box 1:**
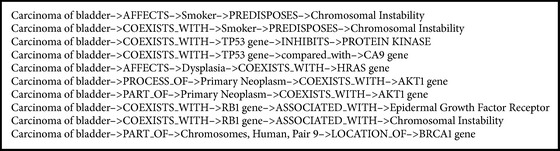
Top 10 predications scored by random walk with depth 4.

**Box 2:**
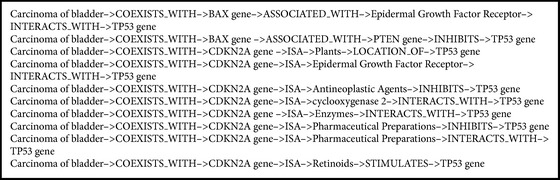
Top 10 predications scored by random walk with depth 6.

**Box 3:**
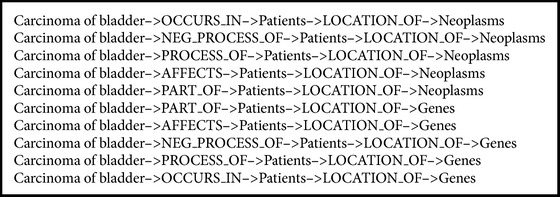
Top 10 predications of cooccurence scored by random walk with depth 4.

**Box 4:**
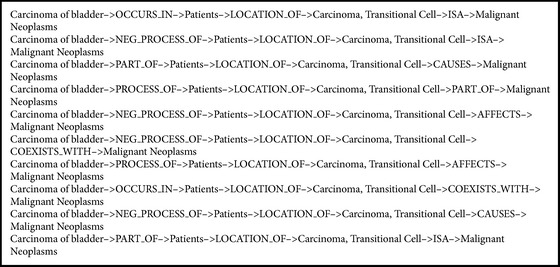
Top 10 predications of cooccurence scored by random walk with depth 6.

**Table 1:** Kullback-Leibler Divergence scores of relations between *Carcinoma of bladder* and genes.

Relation	KLD
ASSOCIATED_WITH	0.264763
PART_OF	0.196826
PREDISPOSES	0.056031
COEXISTS_WITH	0.047783
AFFECTS	0.017736

**Table 2:** Full name for semantic types in disease-gene predication.

Semantic type	Full name
aapp	Amino acid, peptide, or protein
bacs	Biologically active substance
celc	Cell component
cell	Cell
comd	Cell or molecular dysfunction
gngm	Gene or genome
neop	Neoplastic process
tisu	Tissue

**Table 3:** KM scores of predicates and semantic types between *Carcinoma of bladder* and genes.

Semantic type	Relation	KM
gngm	ASSOCIATED_WITH	0.036137
tisu	PART_OF	0.007876
aapp	ASSOCIATED_WITH	0.007778
bacs	ASSOCIATED_WITH	0.004029
comd	COEXISTS_WITH	0.001836

**Table 4:** Combo scores of predicates and semantic types between *Carcinoma of bladder* and genes.

Semantic type	Relation	Combo
tisu	PART_OF	0.355117
gngm	ASSOCIATED_WITH	0.212097
celc	PART_OF	0.208429
aapp	ASSOCIATED_WITH	0.119630
cell	PART_OF	0.098452

**Table 5:** Ranking and recall for genes confirmed with reference standard for IDF results.

Gene	KM ranking	KM analysis	Combo ranking	Combo analysis
TP53	1	TP	1	TP
FGFR3	2	TP	2	TP
HRAS	6	TP	7	TP
TSC1	23	TP	26	TP
MDM2	29	TP	32	TP
RB1	58	TP	70	TP
ERCC2	70	TP	82	TP
NAT2	75	TP	87	TP
RAG1	NULL	FN	NULL	FN
MTCYB	NULL	FN	NULL	FN
ATM	NULL	FN	NULL	FN
TGFB1	NULL	FN	NULL	FN
ERBB3	NULL	FN	NULL	FN
MAP	39.46%		37.52%	

**Table 6:** Ranking and MAP for genes related to Parkinson Disease confirmed with reference standard.

Gene	KM ranking	KM analysis	Combo ranking	Combo analysis
LRRK2	1	TP	3	TP
PARK7	2	TP	7	TP
PINK1	5	TP	11	TP
SNCA	8	TP	14	TP
GIGYF2	9	TP	16	TP
NR4A2	11	TP	28	TP
VPS35	12	TP	24	TP
PARK2	13	TP	25	TP
ATP13A2	20	TP	40	TP
GBA	24	TP	39	TP
UCHL1	NULL	FN	NULL	FN
PRKAG2	NULL	FN	NULL	FN
SNCB	NULL	FN	NULL	FN
PLA2G6	NULL	FN	NULL	FN
PDE8B	NULL	FN	NULL	FN
NDUFAF3	NULL	FN	NULL	FN
FOXRED1	NULL	FN	NULL	FN
NDUFA11	NULL	FN	NULL	FN
NDUFA1	NULL	FN	NULL	FN
NDUFAF2	NULL	FN	NULL	FN
NDUFAF4	NULL	FN	NULL	FN
NDUFAF5	NULL	FN	NULL	FN
NDUFAF6	NULL	FN	NULL	FN
NDUFS1	NULL	FN	NULL	FN
NDUFS2	NULL	FN	NULL	FN
NDUFS4	NULL	FN	NULL	FN
NUBPL	NULL	FN	NULL	FN
SLC6A3	NULL	FN	NULL	FN
DRD4	NULL	FN	NULL	FN
FGF20	NULL	FN	NULL	FN
SNCAIP	NULL	FN	NULL	FN
NDUFV2	NULL	FN	NULL	FN
STX1B	NULL	FN	NULL	FN
NDUFV1	NULL	FN	NULL	FN
HP	NULL	FN	NULL	FN
PARK	NULL	FN	NULL	FN
MAP	62.66%		27.86%	
